# Design and Development of an Interactive Web-Based Simulator for Trauma Training: A Pilot Study

**DOI:** 10.1007/s10916-021-01767-y

**Published:** 2021-09-25

**Authors:** Blanca Larraga-García, Luis Castañeda López, Francisco Javier Rubio Bolívar, Manuel Quintana-Díaz, Álvaro Gutiérrez

**Affiliations:** 1 Escuela Técnica Superior de Ingenieros de Telecomunicación, Universidad Politécnica de Madrid, Avenida de Complutense, 30, 28040 Madrid, Spain; 2 Hospital La Paz Institute for Health Research, IdiPAZ, Madrid, Spain; 3 CEASEC (Centro avanzado de simulación y entrenamiento clínico), Calle de Pedro Rico, 6, 28029, Madrid, Spain; 4 Departamento de Medicina, Facultad de Medicina Universidad Autónoma de Madrid, Calle Arzobispo Morcillo, 4, 28029, Madrid, Spain

**Keywords:** Trauma, Medical education, Training, Simulation, Treatments

## Abstract

Trauma is the leading cause of death in people under 45 years old and one of the leading causes of death in the world. Therefore, specific trauma training during medical school as well as after it is crucial. Web-based learning is an important tool in education, offering the possibility to create realistic trauma scenarios. A web-based simulator has been developed and a pilot study has been accomplished to trial the simulator. A pelvic trauma scenario was created and 41 simulations were performed, 28 by medical students and 13 by doctors. The data analyzed are the actions taken to treat the trauma patient, the evolution of the vital signs of the patient, the timing spent on deciding which action to take, when each action was performed and the consequence that it had on the patient. Moreover, a post-simulation questionnaire was completed related to the usability of the simulator. The clinical treatment performance of doctors is better than the performance of medical students performing more actions correctly and in the right sequence as per ATLS recommendations. Moreover, significant differences are obtained in the time response provided to the patients which is key in trauma. With respect to the usability of the tool, responses provide a positive usability rating. In conclusion, this pilot study has demonstrated that the web-based training developed can be used to train and evaluate trauma management. Moreover, this research has highlighted a different approach to trauma treatment between medical students and doctors.

## Purpose

Trauma is one of the leading causes of death in the world being the main cause of death in people under 45 years old [1]. Trauma deaths have followed a classical trimodal distribution [2], but the epidemiology of these deaths have changed since the year 2000 towards a bimodal distribution in which the third peak is no longer detected [3–5]. Whereas immediate deaths are still quite high, the second and the third peak merge, not showing differences, as deaths tend to constantly decline with time [6, 7].

Therefore, as immediate deaths are still an important number, trauma training remains to be a necessary task. The advanced trauma life support (ATLS) training was created in 1978 in the United States [8] and since then, it has been disseminated all around the globe being the main trauma standard in approximately 44 countries [9]. Nevertheless, this training is only available for doctors and not medical students. Due to this fact, medical students request more trauma specific training [10, 11] and the American College of Surgeons have created an introductory trauma course named Trauma Evaluation and Management (TEAM) [9]. Moreover, the ATLS training is restricted to a number of students per year and therefore, there is a waiting list that depending on the country could vary from 6 months to 2½ years. Taking all this into account, other trauma training methods are arising [12–15]. Some of these trainings focus on prehospital trauma management [16–20], some other center on specific technical skills [21] and, additionally, trauma trainings are incorporating non-technical skills to improve the management of the trauma patient [22–24]. Technical skills pay attention to training specific techniques and treatments whereas non-technical skills put emphasis on managing the trauma scenario including communication, leadership and coordination with different clinical specialties.

The role of simulation in clinical training is key [25–28]. There are several simulation modalities and each of them has advantages and disadvantages [29, 30]. Simulation could be classified as low-fidelity, medium-fidelity and high-fidelity. The fidelity refers to the degree of accuracy with which a simulator represents a real clinical situation. Low-fidelity simulation is used to train specific technical skills such as airway management whereas high-fidelity simulation is used to train non-technical skills including a simulated patient in which the simulator is able to replicate a real clinical scenario. To do so, different simulators are used from high-fidelity mannequins to standardized patients, skill stations or web-based simulation [29, 30].

Web-based simulation allows to provide an authentic learning environment to train experiences that could happen in the clinical practice. Moreover, it allows the possibility to train a high number of students simultaneously providing an objective training tool that is demanded in medical schools [31]. Web-based simulators offer several possibilities providing flexibility to the trainings, allowing several profiles to access to the different trainings and it is more cost-effective than mannequin-based simulation. Moreover, it offers the possibility to objectively evaluate the simulation which is an important aspect considering that there is a lack of reliable objective evaluation tools in emergencies such as a trauma scenario [32].

Thus, the aim of this work is to develop a web-based trauma simulator (WBTS) to train trauma management skills that will allow different profiles to train these skills, evaluate them and easily implement it in any clinical institution.

## Methods

### Web-based simulator

**Fig. 1:**
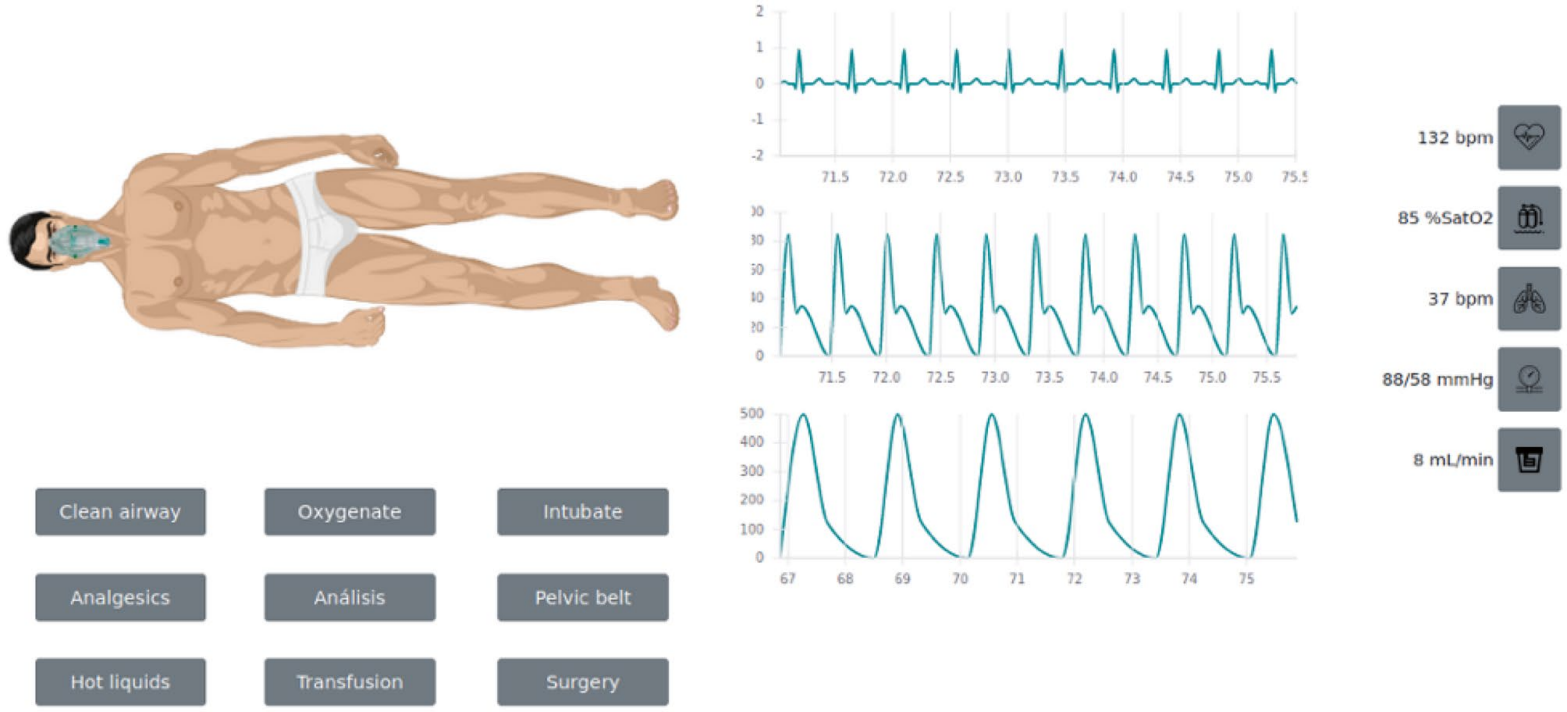
Web-based simulator in which the virtual patient is shown together with his vital signs and some of the actions that could be accomplished

The WBTS developed has as objective to train and support clinicians in the trauma management treatment. Therefore, this tool emulates a virtual patient who suffers a specific traumatic lesion which will be called trauma scenario. This trauma scenario is previously created by a trainer who will be in charge of defining all the details of the trauma scenario to train. Once the trauma scenario is created, it is assigned to a trainee who will be in charge of treating and managing the virtual patient. The main page of the WBTS is composed by three components: the virtual patient, the vital signs of the patient and the actions to accomplish to treat the patient as shown in Fig. 1. Additionally, along the simulation, all the actions accomplished as well as the impact that those actions have on the vital signs of the patient and the timing in which those actions are done are recorded. This allows to issue a report automatically once the simulation is finished in which all this information is shown. This will help the trainee to analyze the simulation in detail and the trainer to objectively evaluate the performance of the trauma scenario. To develop this WBTS, the application created has been designed taking into account three parts: the backend, the frontend and its deployment in Docker [33]. To be able to do so, the following computing environments need to be installed: Node.js, MySQL, Docker and Visual Studio Code. The source code is available at: https://github.com/Robolabo/trauma-simulator.

### Simulation details

The ATLS separates the assessment of a trauma patient care in two parts, a primary and a secondary survey [34]. In the primary survey, life-threatening injuries are managed whereas other injuries are diagnosed and treated in the secondary survey. Therefore, the pilot study that will prove the WBTS will be based on the primary survey of a pelvic trauma scenario. The pelvic trauma scenario has been simplified as no other injury is simulated in the patient.

**Fig. 2:**
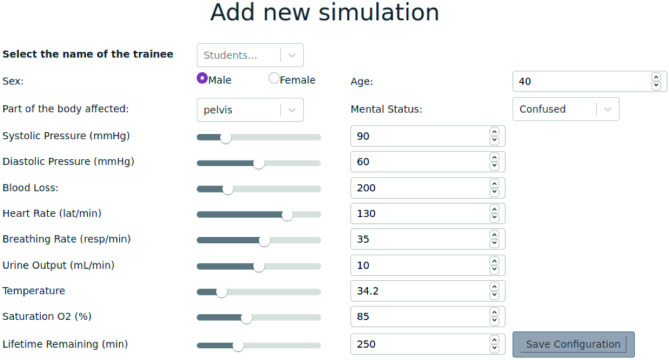
Trauma scenario definition screen. This section is only available for trainers in which the trauma scenario together with the trainee will be defined and selected

The pilot study was conducted at IdiPAZ—Hospital La Paz Institute for Health Research in Madrid, Spain [35]. A pelvic trauma scenario was defined in which the trainer defined the characteristics of the trauma case that the trainee will manage. Therefore the sex of the patient, age, part of the body affected together with the vital signs of the patient at the time of the trauma scenario will be provided as shown in Fig. 2 in which all the details that the trainer sets are shown.

Moreover, the remaining lifetime of the patient if no action is taken is provided. This creates a more realistic scenario in which a fast and efficient response should be provided under stressful circumstances.

Considering the set of actions defined that could be accomplished in the pelvic trauma scenario together with the ATLS guidelines, 432 different sequences of actions could be accomplished to treat the virtual patient and will be determined as references. These different sequences, scenarios, are defined taking into account the different actions to accomplish and the evolution of the virtual patient and have the number of actions shown in Table 1. Therefore, as an example, 12 different scenarios have eight actions which means that, taking into account the actions included in the simulator, 12 different possible scenarios could be performed to treat the patient carrying out eight different actions. These are the scenarios that were possible to accomplish when treating the trauma patient. Each of the participants applied a different procedure, performing a different sequence of actions from all these options. These data were gathered and analyzed.

**Table 1:** Number of scenarios and the number of actions included in each scenario

Number of scenarios	Number of actions
12	8
48	9
96	10
120	11
96	12
48	13
12	14

### Participants

Final-year medical students and doctors with an average experience of 12.36 ± 7.45 years were invited to participate in the pilot study to test the WBTS. An explanation on the WBTS was provided together with the instructions they must follow during the simulation. All participants received a 15 min explanation on the WBTS together with a first trial to get familiar with the simulation set-up. Once the trauma scenario was finished, a post-simulation questionnaire was distributed in order to gather information about the user experience. In total, 28 simulations from final-year medical students and 13 from doctors were analyzed.

### Data analysis

The WBTS has a database associated due to the need to gather and analyze the data generated during the simulation. From one side, this allows the trainee to use this tool as a training tool having access to what has happened during the simulation. One the other side, this allows the trainer to objectively evaluate the performance of a trainee.

The data analyzed are the actions taken to treat the trauma patient, the evolution of the vital signs of the patient, the timing spent on deciding which action to take, when each action was performed and the consequence that it had on the patient. This data is studied to obtain information about which actions were performed during the simulation, if those actions were performed following the ATLS guidelines and if the timing in which the actions were done was the right one for the patient. Moreover, this analysis is done comparing two different groups of participants: final-year medical students and doctors and the Wilcoxon rank-sum test has been used to compare the two samples. This test has been performed in Python and statistical significance is obtained if the p-value is lower than 0.05.

Once the simulation is finished, post-simulation questionnaires are also analyzed to evaluate the usability of the training tool. The questionnaire consists of a set of closed-questions concerning their attitude and perception of the learning experience on a seven-point Likert-type scale ranging from strongly disagree to strongly agree [36]. The questions addressed are shown in Table 2.

**Table 2:** Questions of the usability questionnaire

Questions
Q1. In general, the tool is easy to use
Q2. I feel comfortable with the tool
Q3. The simulator is easy to learn
Q4. The tool shows errors and how to solve them
Q5. The information provided in the simulator is clear
Q6. It is easy to find the information
Q7. The information provided is easy to understand
Q8. The information provided is effective to support the simulation
Q9. The organization of the information in the screen is clear
Q10. Do you consider the order of the elements of the screen adequate?
Q11. Do you consider that all the needed parameters/monitoring information to treat a pelvic trauma scenario are shown in the simulator?
Q12. The interface is friendly
Q13. I like the interface of the simulator
Q14. The simulator has all the functionalities that you would like to have in a web-based simulator
Q15. In general, you feel satisfied with the simulator

## Results

The WBTS was tested with the pelvic trauma case explained. All the participants completed successfully the trial and a comparison analysis between the performance of the final-year medical students and doctors was done.

From all the data gathered several parameters were analyzed as shown in Table 3.

**Table 3:** Parameters analyzed during the simulations and comparison between medical students and doctors

Parameter	Medical students	Doctors
Treatment time	237 ± 68 (mean ± SD)	228 ± 62 (mean ± SD)
Number of actions performed	10 ± 2 (mean ± SD)	10 ± 2 (mean ± SD)
Number of correct actions performed	7 ± 6 (mean ± SD)	7 ± 4 (mean ± SD)
Number of sequential actions performed	3 ± 2 (mean ± SD)	4 ± 3 (mean ± SD)
Airway inspection	71% (20/28)	85% (11/13)
Patient oxygenation	96% (27/28)	100% (13/13)
Patient intubation	68% (19/28)	54% (7/13)
Pelvic binder placement	71% (20/28)	77% (10/13)
Blood transfusion	86% (24/28)	92% (12/13)
Crystalloids administration	71% (20/28)	85% (11/13)
Thermal blanket	43% (12/28)	15% (2/13)
Hot liquids administration	21% (6/28)	62% (8/13)

Some of the parameters show no difference between the groups. For example, the timing spent on the treatment in both groups is around 230 min. Both groups perform a mean of seven correct actions along the treatment. Taking into account the 432 different sequences of treatment, these results show that in some cases (14% of them) seven actions are close to the number of actions needed to treat the patient as shown in Table 1. However, in most of the cases (86%) they are not enough. Additionally, the correct sequence of actions taken to treat the patient as per ATLS guidelines is analyzed. Moreover, the mean of correct sequential actions as per ATLS is three actions for students whereas four for doctors, showing no significant differences. However, some other variables are observed to report important differences between the groups. They are explained hereafter.

### Airway assessment

**Fig. 3:**
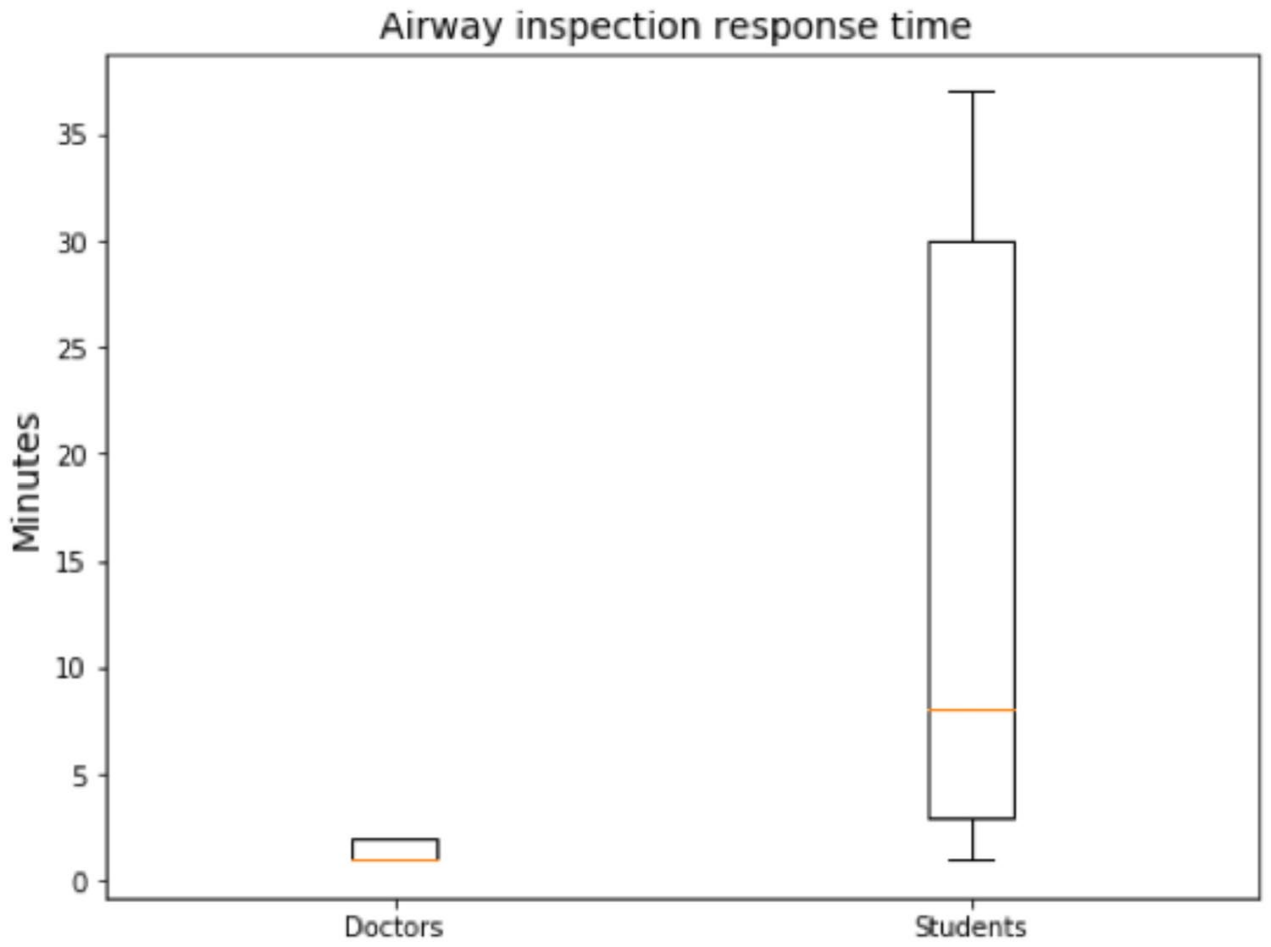
Airway inspection response and the differences between doctors and students

The first step to accomplish during the primary survey of a trauma patient is the airway assessment. 71% of the students takes this first step whereas only 84% of the doctors does it. From the students that inspect the airway, they do it from the third minute of treatment onward whereas the doctors start to inspect the airway during the first two minutes, showing significance (p-value = 0.0020) between the two groups analyzed as shown in Fig. 3.

### Breathing

**Fig. 4:**
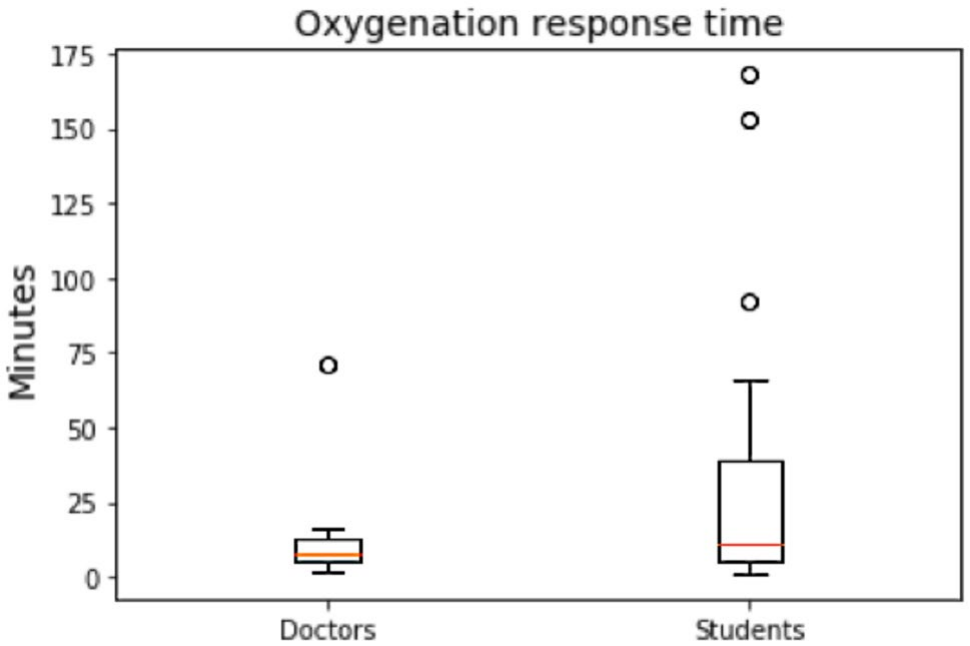
Oxygenation response time performed by doctors and by medical students

Breathing is the second item to accomplish during the primary survey. As in this case there are no pneumothorax, hemothorax or contusions, the clinical problems that may arise with respect to assure a correct breathing of the patient could be treated with measures such as ventilation or intubation. Actually, 96% of the students oxygenate the virtual patient and all the doctors also do it. The difference between the two groups is the moment in time in which they oxygenate the patient but not statistical significance is obtained (p-value = 0.5129). Students oxygenate in a wider range and in moments in time that are quite late such as 150 or 166 min after the arrival of the patient to the medical facility. All the doctors do it more or less in a similar time range as shown in Fig. 4.

### Circulation and hemorrhage control

**Fig. 5:**
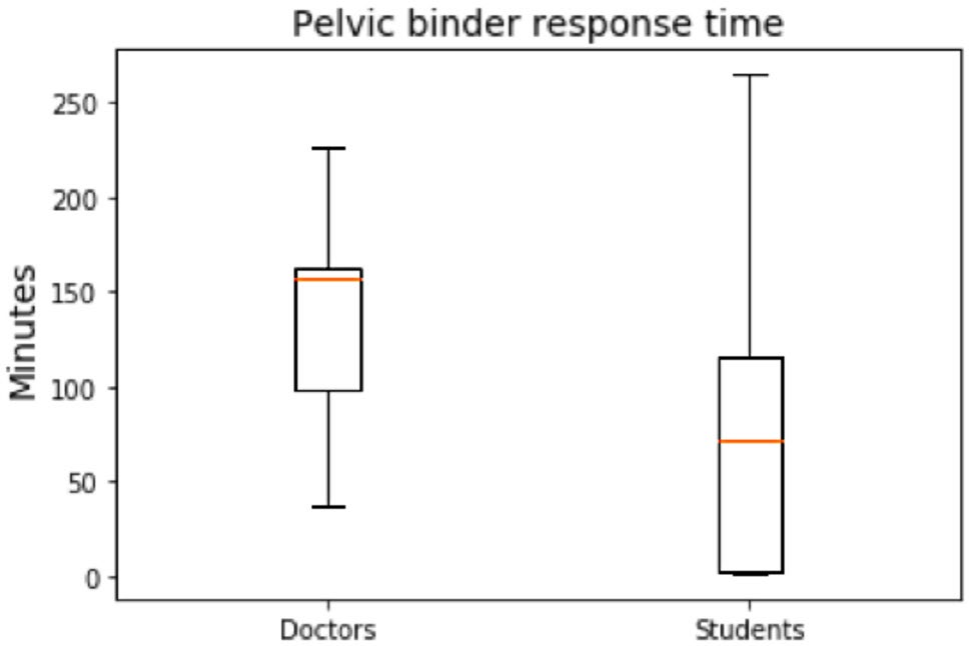
Response time in placing a pelvic binder to the patients for both doctors and students

Circulation is the third priority during the primary survey. Notice that the main cause of problems in circulation are hemorrhages. Because the virtual patient suffers a pelvic trauma, a pelvic binder should be placed as soon as possible in order to decrease blood loss. Then, liquids should be provided in order to reinstate a normal blood volume as soon as possible. 71% of the students place a pelvic binder on the patient and 77% of the doctors does it. Nevertheless, the response in time of the location of this device is done late in both groups as shown in Fig. 5 and statistical significance is obtained (p-value = 0.0165). The median response time of the students is 72 min [Q1 = 2.50, Q3 = 116] and for the doctors it is 157 min [Q1 = 99, Q3 = 162.75]. Therefore, students perform this action better than doctors but, in both cases, a scarcity is trauma training is clearly perceived.

Moreover, 86% of the students infuse liquids to regain a normal blood volume and 92% of the doctors does it too to support the blood circulation restoration.

### Disability

Disability should be assessed as the fourth task to accomplish during the primary survey. None of the participants made this assessment nor the students nor the doctors.

### Exposure

The last step of the primary survey considers the exposure of the patient such as hypothermia, burns or possible exposure to chemicals. In the cases in which the body temperature of the patient is below or equal to 35 °C, 64% of the students provides the patient with hot liquids or with a thermal to increase the temperature of the patient. The percentage of doctors that take those measures in order to increase the body temperature of the patients is of 77%. There have been no important differences between both groups with respect to when the trainees, students or doctors, apply a treatment to the virtual patient to avoid hypothermia. The patient of this trauma scenario does not suffer any burns or exposure to chemicals.

### Usability of the web-based simulator

**Fig. 6:**
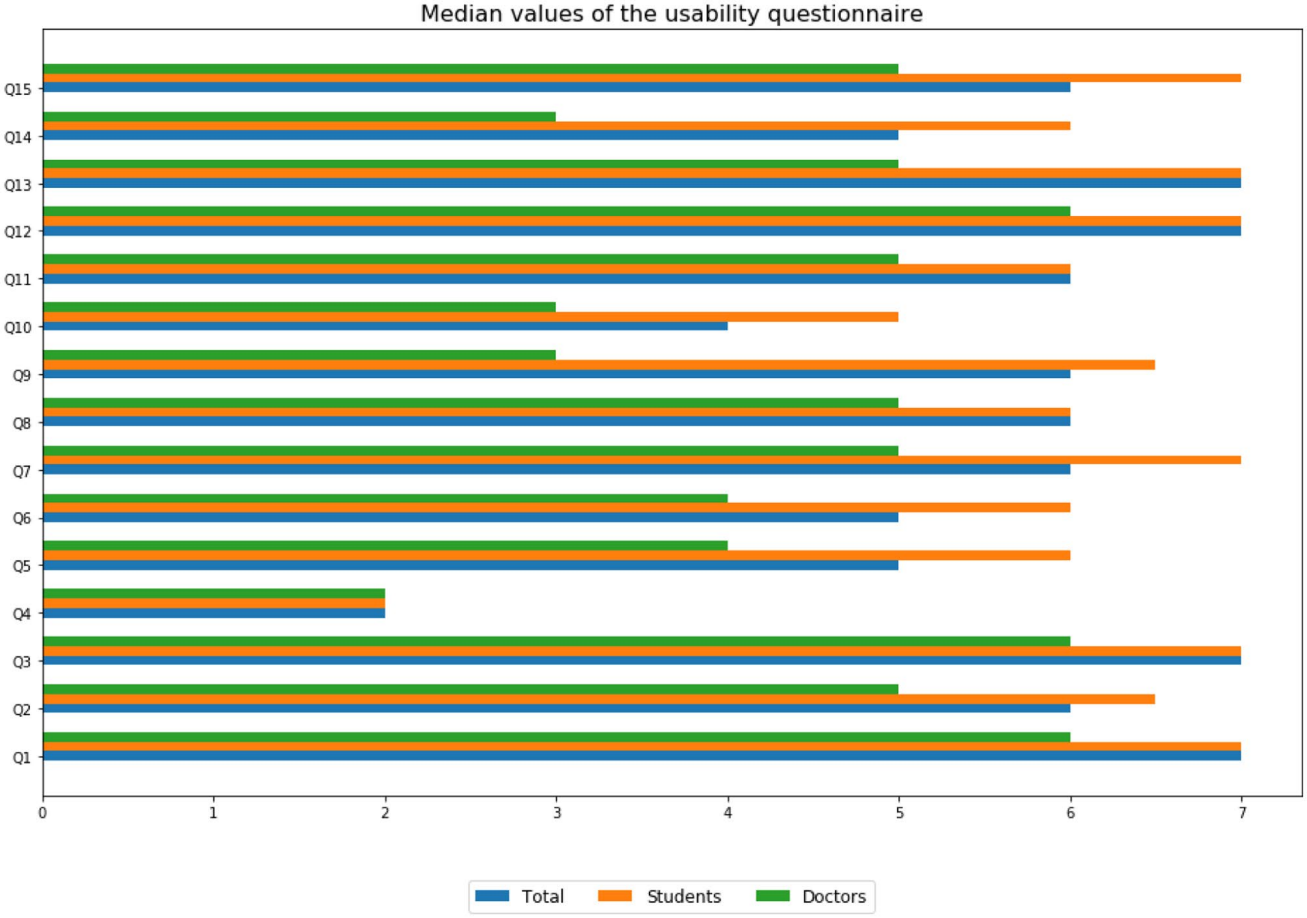
Usability post-simulation questionnaire. The values shown are the medians of the answers provided by all the participants in blue and by the students in orange and the doctors in green (colour figure online)

A post-simulation questionnaire was provided to all the participants in order to evaluate the usability of the WBTS developed. This questionnaire aims to evaluate the usability of the tool in which seven is the highest mark and one is the lowest one. In all the questions addressed as shown in Fig. 6, the doctors provide a lower score than medical students except for question Q4. This question is the one that refers to “The tool shows errors and how to solve them”.

Four questions get the maximum median punctuation in total, six questions get the second maximum median punctuation. Three questions get a five and only one question gets a lower score which is Q4.

## Discussion

The goal of this pilot study was to demonstrate that the WBTS can be used to train and evaluate trauma management. Taking into account the results presented in the previous section, this goal is achieved and a clear need for trauma management is detected taking into account the results obtained. The number of correct actions performed to treat the trauma patient were not enough in the majority of the cases and as well as the number of correct sequential actions accomplished. Both groups, students and doctors, showed a need for training being more noticeable in the medical students group. There are some trauma trainings to medical students but the best practice to teach trauma management at an undergraduate level has not yet been determined [30, 37]. Additionally, with this web-based simulation, it is possible to gather objective information of all the steps taken during the simulated trauma scenario which matches with one of the objectives of clinical simulation: to allow a more objective evaluation of a simulated scenario [38, 39]. This has a positive impact, not only on the evaluation process, but also on the learning process of the trainees. They will have all the information to analyze what they have done and what has happened during the trauma scenario. Moreover, using the web-based technology allows the trainees to repeat the simulation as many times as needed offering the possibility to train several trainees at the same time.

### Airway assessment

It is expected that differences appear between the two groups, medical students versus doctors. Actually, this is the action in which the difference between the two groups is clearer. Moreover, it is critical for a trauma patient to inspect first of all the airway in order to clean it in case it is necessary. If this is not done at the very beginning, the life of the patient might be in serious danger. Therefore, there is a clear need to train students on trauma treatment as only half of the students inspect the airway along the treatment and all of them do it in a timing which is not adequate for a trauma patient.

### Breathing

With respect to breathing, there are also important differences. In this case almost all of the students performed this second item to accomplish during the primary survey but most of them do it late in time. Oxygenation is key to bring oxygen to tissues and organs and if this is not guaranteed important consequences on the patient’s life might happen. Training students not only on performing this task but also on when to do it is necessary to avoid important consequences that might risk the life of a patient with a trauma lesion.

### Circulation and hemorrhage control

With respect to hemorrhage control and blood volume restoration, it seems that more training is needed with respect to the use of a pelvic binder and when to use it. The pelvic binder is key to control hemorrhages and should be placed as soon as possible, if not, tasks such as providing crystalloids of blood transfusions will not be of value as the volume infused will be lost through the hemorrhage; therefore, for both students and doctors it is important to train pelvic trauma protocols to reinforce the importance of this task and when to accomplish it.

### Disability

Even though the WBTS offers the option to talk to the patient to find it how he or she feels, none of the participants used this option focusing on the rest of actions that they may accomplish during the simulated trauma scenario. In this case, the patient was confused after the lesion, but no dialogue was started, and no specific procedure was to evaluate the visual, verbal and motor response of the patient.

### Exposure

To pay attention to other important aspects to which the patient might be exposed is also important. In this case, the patient temperature was below 35 °C in most of the cases taking into account the evolution of the patient and the actions taken. To increase the body temperature is a must. This pilot study showed that both groups, students and doctors have to payed more attention to this fact as there is still an important percentage of trainees that did not take any measure.

### Usability of the web-based simulator

With respect to the usability of the tool, the responses were all quite positive. There are some aspects to improve such as the information provided in the tool and how it was shown. The actions could be grouped facilitating the treatment of the patient and this might be done taking into account the profile of the trainee. Also, more functionalities could be included providing more options to interact with the virtual patient and allowing to explore the patient. Nevertheless, the satisfaction of the WBTS was high.

## Conclusion

The pilot study accomplished demonstrates that the WBTS is suitable to train and evaluate trauma management. The simulator allows to interact with a virtual patient as the treatments applied have an impact on the patient. Moreover, all the treatments are recorded as well as the impact on the vital signs of the patient which allows the trainee to use this simulator as a learning tool and it allows the trainer to objectively evaluate the performance of the trainee.

Additionally, this study has highlighted the scarcity trauma training specially in the medical students’ community as highlighted in [10, 11]; nevertheless, also doctors may need trauma management refreshing courses [40]. The skills gained from an ATLS training need to be reinforced. If they are not maintained, they start to decrease after six months [41–44]. Therefore, a WBTS could be implemented to further refresh the main trauma management skills. Moreover, it has been proved that simulation training programs may have an impact on decreasing the time for trauma treatment [45–47]. Consequently, this WBTS presents an alternative tool to continue improving the treatment time.

Nonetheless, further work needs to be done implementing more trauma scenarios that would allow to cover more lesions as well as polytrauma patients. Also, the pilot study should be extended to a larger community performing a regular training using this tool and analyzing its impact. To do so, the tool should be used for a predefined period between the first simulation case and the last one. The students evolution should be analyzed in order to assess the impact of the WBTS on trauma management learning. Additionally, a specific debriefing session should be developed to provide a comprehensive analysis of the simulations performed.

## Data Availability

https://github.com/Robolabo/trauma-simulator

## References

[1] Trauma Statistics & Facts—Coalition for National Trauma Research. Coalition for National Trauma Research. https://www.nattrauma.org/trauma-statistics-facts/. Published 2020. Accessed December 15, 2020.

[2] Trunkey D, Lim R (1974). Analysis of 425 consecutive trauma fatalities: An autopsy study. Journal of the American College of Emergency Physicians..

[3] Kleber C, Giesecke M, Tsokos M (2012). Overall Distribution of Trauma-related Deaths in Berlin 2010: Advancement or Stagnation of German Trauma Management?. World J Surg..

[4] Pang J, Civil I, Ng A, Adams D, Koelmeyer T (2008). Is the trimodal pattern of death after trauma a dated concept in the 21st century? Trauma deaths in Auckland 2004. Injury..

[5] Evans J, van Wessem K, McDougall D, Lee K, Lyons T, Balogh Z (2009). Epidemiology of Traumatic Deaths: Comprehensive Population-Based Assessment. World J Surg..

[6] Rauf R, von Matthey F, Croenlein M (2019). Changes in the temporal distribution of in-hospital mortality in severely injured patients—An analysis of the TraumaRegister DGU. PLoS One..

[7] Lansink K, Gunning A, Leenen L (2013). Cause of death and time of death distribution of trauma patients in a Level I trauma centre in the Netherlands. European Journal of Trauma and Emergency Surgery..

[8] American College of Surgeons Committee on Trauma (2018). Advanced trauma life support program for doctors.

[9] Advanced Trauma Life Support. American College of Surgeons. https://www.facs.org/Quality-Programs/Trauma. Published 2020. Accessed December 15, 2020.

[10] Jouda M, Finn Y (2020). Training in polytrauma management in medical curricula: A scoping review. Med Teach..

[11] Mastoridis S, Shanmugarajah K, Kneebone R (2011). Undergraduate education in trauma medicine: The students’ verdict on current teaching. Med Teach..

[12] Wallin C, Meurling L, Hedman L, Hedegård J, Felländer-Tsai L (2007). Target-focused medical emergency team training using a human patient simulator: effects on behavior and attitude. Med Educ..

[13] Minor S, Green R, Jessula S (2019). Crash testing the dummy: a review of in situ trauma simulation at a Canadian tertiary centre. Canadian Journal of Surgery..

[14] Long A, Mowery N, Chang M (2015). Golden Opportunity: Multidisciplinary Simulation Training Improves Trauma Team Efficiency. J Am Coll Surg..

[15] Harrington C, Kavanagh D, Quinlan J (2018). Development and evaluation of a trauma decision-making simulator in Oculus virtual reality. The American Journal of Surgery..

[16] Mobrad A, Al Najjar A, Abu Zeid R, Atta Aldayes A (2020). Evaluating the effect of the prehospital trauma life support (PHTLS) course on emergency medical services students? knowledge. Biomedical Research.

[17] Häske D, Beckers S, Hofmann M (2017). Quality of Documentation as a Surrogate Marker for Awareness and Training Effectiveness of PHTLS-Courses Part of the Prospective Longitudinal Mixed-Methods EPPTC-Trial. PLoS One..

[18] Ali J, Adam R, Josa D (1998). Effect of Basic Prehospital Trauma Life Support Program on Cognitive and Trauma Management Skills. World J Surg..

[19] Requena A, Jiménez L, Gómez R, del Arco C (2015). International Trauma Life Support (ITLS) training through the Spanish Society of Emergency Medicine (SEMES): 10 years' experience with the SEMES-ITLS program. Emergencias..

[20] Campbell J, International Trauma Life Support (ITLS) (2013). International Trauma Life Support For Emergency Care Providers.

[21] Kuhlenschmidt K, Houshmand N, Bisgaard E (2020). Simulation-Based Skill Training in Trauma: A Much Needed Confidence Boost. J Am Coll Surg..

[22] Ziesmann M, Widder S, Park J (2013). S.T.A.R.T.T.. Journal of Trauma and Acute Care Surgery..

[23] Gillman L, Brindley P, Paton-Gay J (2016). Simulated Trauma and Resuscitation Team Training course? evolution of a multidisciplinary trauma crisis resource management simulation course. The American Journal of Surgery..

[24] Doumouras A, Engels P (2017). Early crisis nontechnical skill teaching in residency leads to long-term skill retention and improved performance during crises: A prospective, nonrandomized controlled study. Surgery..

[25] Abelsson A, Rystedt I, Suserud B, Lindwall L (2014). Mapping the use of simulation in prehospital care—a literature review. Scand J Trauma Resusc Emerg Med..

[26] Murray D, Freeman B, Boulet J, Woodhouse J, Fehr J, Klingensmith M (2015). Decision Making in Trauma Settings. Simulation in Healthcare: The Journal of the Society for Simulation in Healthcare..

[27] Van Dillen C, Tice M, Patel A (2016). Trauma Simulation Training Increases Confidence Levels in Prehospital Personnel Performing Life-Saving Interventions in Trauma Patients. Emerg Med Int..

[28] Cuisinier A, Schilte C, Declety P (2015). A major trauma course based on posters, audio-guides and simulation improves the management skills of medical students: Evaluation via medical simulator. Anaesthesia Critical Care & Pain Medicine..

[29] Quick JA (2018). Simulation Training in Trauma. Mo Med..

[30] Borggreve A, Meijer J, Schreuder H, ten Cate O (2017). Simulation-based trauma education for medical students: A review of literature. Med Teach..

[31] Wise E, McIvor W, Mangione M (2016). Assessing student usage, perception, and the utility of a Web-based simulation in a third-year medical school clerkship. J Clin Anesth..

[32] Murray D, Boulet J, Ziv A, Woodhouse J, Kras J, McAllister J (2002). An acute care skills evaluation for graduating medical students: a pilot study using clinical simulation. Med Educ-..

[33] Arsys. What is docker and which are the advantages of working with its containers? https://www.arsys.es/blog/soluciones/docker-ventajas-contenedores/, 2019. [Online]; Accessed 29 May 2020.

[34] Driscoll P, Gwinnutt C, McNeill I (1999). Controversies in advanced trauma life support. Trauma..

[35] IdiPAZ—Instituto de Investigación Hospital Universitario La Paz. Idipaz.es. https://www.idipaz.es/DefaultEN.aspx. Published 2020. Accessed 15 December 2020.

[36] Korkut Altuna O, Arslan F (2016). Impact of the Number of Scale Points on Data Characteristics and Respondents’ Evaluations: An Experimental Design Approach Using 5-Point and 7-Point Likert-type Scales. İstanbul Üniversitesi Siyasal Bilgiler Fakültesi Dergisi.

[37] Jouda M, Finn Y (2020). Training in polytrauma management in medical curricula: A scoping review. Med Teach..

[38] Ali J, Dunn J, Eason M, Drumm J (2010). Comparing the Standardized Live Trauma Patient and the Mechanical Simulator Models in the ATLS Initial Assessment Station. Journal of Surgical Research..

[39] Wallenstein J, Heron S, Santen S, Shayne P, Ander D (2010). A Core Competency-based Objective Structured Clinical Examination (OSCE) Can Predict Future Resident Performance. Academic Emergency Medicine..

[40] Wong K (2007). Trauma education of junior hospital doctors at a major Australian trauma service. The Surgeon..

[41] Mohammad A, Branicki F, Abu-Zidan F (2013). Educational and Clinical Impact of Advanced Trauma Life Support (ATLS) Courses: A Systematic Review. World J Surg..

[42] Ali J, Cohen R, Adam R (1996). Attrition of Cognitive and Trauma Management Skills after the Advanced Trauma Life Support (ATLS) Course. The Journal of Trauma: Injury, Infection, and Critical Care..

[43] Ali J, Howard M, Williams J (2002). Is attrition of advanced trauma life support acquired skills affected by trauma patient volume?. The American Journal of Surgery..

[44] Ali J, Howard M, Williams J (2003). Do Factors Other Than Trauma Volume Affect Attrition of ATLS-Acquired Skills?. The Journal of Trauma: Injury, Infection, and Critical Care..

[45] Park C, Grant J, Dumas R (2020). Does simulation work? Monthly trauma simulation and procedural training are associated with decreased time to intervention. Journal of Trauma and Acute Care Surgery..

[46] Murphy M, Curtis K, Lam M, Palmer C, Hsu J, McCloughen A (2018). Simulation-based multidisciplinary team training decreases time to critical operations for trauma patients. Injury..

[47] Long A, Lefebvre C, Masneri D (2019). The Golden Opportunity: Multidisciplinary Simulation Training Improves Trauma Team Efficiency. J Surg Educ..

